# Modeling sepsis using neural nets and biomarkers of organ dysfunction in patients, with links to animal models

**DOI:** 10.1186/cc11688

**Published:** 2012-11-14

**Authors:** J Schentag, D Parish, S Opal

**Affiliations:** 1 University at Buffalo, Buffalo, NY, USA; 2 CPL Associates LLC, Buffalo, NY, USA; 3 Brown University, Providence, RI, USA

## Background

Most clinical trials of sepsis treatment modalities fail at their primary objective of establishing superiority over placebo when added to background standard of care. While there is no definitive explanation for the high failure rate, it might be stated that our attempts to insert a new therapeutic agent into standard of care encounters severe problems with definition of exactly what stage is ongoing, and what are the criteria for progression or resolution from that time point onwards. Clearly there is need for a means of defining steps in the septic process that would apply to individuals, and to better define the course of sepsis in each patient after they are enrolled in a trial.

## Methods

For core model development, 30 septic patients were studied for time-related progression in relation to biomarkers, employing a Load Model in a neural net algorithm in MatLab. Causative bacterial infections were linked to primary infection sites. In order to minimize over-parameterization, the model was allowed to estimate outputs using the best three input parameters. Bacterial load was tracked from origin using clinical and microbiologic data to provide an estimate at the start of sepsis. The bacterial load as well as clinical and laboratory parameters were model inputs with the output parameter being organ failures and/or mortality.

## Results

At onset of sepsis, human bacterial load estimates ranged from between 10^8 ^and 10^11 ^CFU, which is consistent with inocula in animal models of sepsis. Sepsis proceeds to organ failures and mortality in a series of steps that are initially linked to bacterial load and inflammatory response, followed by coagulopathy, ischemia, oxygen deprivation in organs and tissues, and culminating in organ failures. The later stages of sepsis are all driven by metabolic parameters, and there seems to be little benefit to blocking inflammation at later stages. Substrate and oxygen deficiencies must be addressed first.

## Conclusion

Neural net progression models based on biomarkers and physiological markers are able to describe the evolution of sepsis to septic shock, organ failures, and provide some evidence that mortality may be a consequence of the stages of sepsis. Overall, these models appear useful to the task of sorting out organ failure endpoints and mechanisms in individual patients with sepsis progression across sepsis to septic shock.

